# Supplementary material for: A peptide tag-specific nanobody enables high-quality labeling for dSTORM imaging
Source: Nat Commun. 2018 Mar 2;9:930. doi: 10.1038/s41467-018-03191-2 (PMC5834503; doi:10.1038/s41467-018-03191-2)
Supplement: Supplementary file 1 — Supplementary Information [file 41467_2018_3191_MOESM1_ESM.pdf]

Supplementary Information

A peptide tag-specific nanobody enables high-quality labeling for dSTORM imaging

Virant et al.,

Supplementary Note 1

As an additional, independent measure, we applied Fourier-Ring Correlation (FRC)[1]. The Fourier Image Resolution (FIRE) value is determined by the highest spatial frequencies which still positively correlate with each other above a chosen threshold. Thus, in the case of highly blinking dyes like AF647, the FIRE value mainly correlates with the optical resolution. Only when using a blinking correction factor or in the case of seldom-blinking fluorophores like PAmCherry it also takes the structural resolution into account[1]. Since all nanobodies were coupled to AF647, their stainings yielded highly similar FIRE values of 40 to 50 nm. These values correlate with the 9 to 12 nm NeNA localization precisions, which also mainly rely on the optical properties of the fluorophore (**Supplementary Fig. 6**). Notably, the labeling coverages of the individual fibers do not correlate with their NeNA and FIRE values which illustrates why the optical resolution should not be mistaken as an overall resolution. In the case of PAmCherry, a relatively seldom-blinking fluorophore, blinking events have a much smaller impact on the calculation of the FIRE value. Consequently, FIRE values for PAmCherry are closer to the actual overall resolution as the structural resolution is taken into account. As PAmCherry has low coverages of 35% for the thinnest fibers and up to 75% for the thickest fibers, the high FIRE values of 120 nm clearly illustrates that the limiting factor for the overall resolution is not the optical resolution (NeNA 17 nm) but rather the low coverage (**Supplementary Fig. 6**). The contribution of the linkage error to overall resolution is shown by comparing the sizes of the thinnest filaments stained with either nanobodies or conventional antibodies (**Supplementary Fig. 7**). The differences cannot be differentiated using the FIRE value since both approaches use AF647 as the readout (**Supplementary Fig. 7**). The bias in FIRE values is also visible in the structural analysis of $_{BC2T}$actin filaments (**Supplementary Fig. 9b**).

Supplementary Table 1

Primer sequences

primer name	Sequence (5´- 3´)
PAmCherry-F	ATATATACCGGTGCCACCATGGTGAGCAAGGGCGAGG
PAmCherry-R	ATATATAGATCTGCTTGTACAGCTCGTCCATGCC
VIM-BC2T-for	ATATATGCTAGCGCCACCATGTCCACCAGGTCCGTGTCCTCGTCC
VIM-BC2T-rev	GATCCGGTGGATCCCGGGCCC
VIM-for	AGTGAACCGTCAGATCCGCTA
VIM-rev	TCCACCTAAGCTTGAGCTCGAGATCTGTTCAAGGTCATCGTGATGCT
PAmCherry/eGFP-for	TCGAGCTCAAGCTTAGGTGGAGGAGGTTCTGTGAGCAAGGGCGAGGA
PAmCherry/eGFP-rev	ATCTAGAGTCGCGGCCGCTTACTTGTACAGCTCGTCCATGC
BC2TActb(1)-for	AAGCGCGCTGTTAGTCACTGGCAGCAAGATGATGATATCGCCGCGCT
BC2TActb(1)-rev	GACTTTCCACACCTGGTTGCTGA
BC2TActb(2)-for	TCAGCAACCAGGTGTGGAAAGTC
BC2TActb(2)-rev*	CGGCGCGCTTTCTGTCTGGCATGGTGGCGACCGGTAGC
BC2TLamin-for	GGACTCGAGATGCCAGACAGAAAGGCGGCTGTTAGTCACTGGCAGCAAGCGACTGCGACC CCCGT
BC2TLamin-rev	GGAGCTAGCATTACATAATTGCACAGCTTCTATTGGAT
laminBC2T-for	AAAGCTAGCGCCACCATGGCGACTGCGACC
laminBC2T-rev*	AAAGCGCGCTTGCGATCAGGCATAATTGCACAGCTTCTA
tubulinBC2T-for	AAAGCTAGCGCTACCGGTCGCCACCATGCGTGAGTGCATCTCCAT
tubulinBC2T-rev*	AAAGCGCGCTTGCGATCAGGGTATTCCTCTCCTTCTTCCTCACCCTC
BC2Ttubulin-for*	AATGCGCGCCGTGAGCCATTGGCAGCAGCGTGAGTGCATCTCCATCC
BC2Ttubulin-rev	ATTGGATCCCTAGTATTCCTCTCCTTCTTCCTCA
BC2TLC3B-for*	GTAGCGCGCCGTGAGCCATTGGCAGCAGCCGTCGGAGAAGACCTTCAA
BC2TLC3B-rev	GGTGGATCCTTACACTGACAATTTCATCCCGA
BC2TYpet-for*	AAAGCGCGCTTGCGATCAGGCATGGTGGCGACCGGTG
BC2TYpet-rev*	AAAGCGCGCCGTCTCTCATTGGCAGCAGGTGAGCAAAGGCGAAGAGCTG
SorTag Ins_for	TTACCGGTCACCACCATCACCATCACTAAG
SorTag Ins_rev	TTACCGGTTTCCGGCAGGCTACCTGAGGAGACGGTGACCTGG
F_KanR_BC2	AAGGCCGCAGTTTCACATTGGCAACAATAAGGCGCGCCAGATCTACTT
R_KanR	GACAGCAGTATAGCGACCAGC
F1_cbp1	ATCAAATTGCTTCGCAGTACATGG
cbp1_BC2_R1	CAATGTGAAACTGCGGCCTTTCTGTCAGGGGTGCTTCTCAAACGAGAAAGATTC
F2_cbp1	AATGCTGGTCGCTATACTGCTGTCTGTCTGTATTCGTTGTGCATATTTGAC
R2_cbp1	GCTCGTATAGCGATTTTGCGTT
ACTB_sgRNA	CTTGTGGAAAGGACGAAACACCCGAGAATAGCCGGGCGCGCTGTTTGGGTCTTCGAGAAG ACCTCACCGCCGTTGTCGACGACGAGCGGTTTTAGAGCTAGAAATAGC
ACTB_HDR	GAAGTGGCCAGGGCGGGGGCGACCTCGGCTCACAGCGCGCCCGGCTATTCTCGCAACTC ACCATGCCTGATCGGAAGGCCGCCGTGAGCCATTGGCAGCAGGATGATGATATCGCCGCG CTCGTCGTCGACAACGGGTCCGGCATGTGCAAGGCCGGCTTCGCGGGCGACGATGCCCC CC
sgRNA_fw	TTTCTTGGCTTTATATATCTTGTGGAAAGGACGAAAC
sgRNA_rev primer	GACTAGCCTTATTTTAACTTGCTATTTCTAGCTCTAAAAC
ACTB_fw	GGGGCTGGGAATTGGCGCTAATTG
BC2_rev	TGCTGCCAATGGCTCACGGCG

* to facilitate cloning, primer sequence encodes an amino acid substitution A>R at position 5 of BC2-tag. This substitution does not affect binding properties of BC2-Nb as shown previously.[2]

Supplementary Figures

Supplementary Figure 1

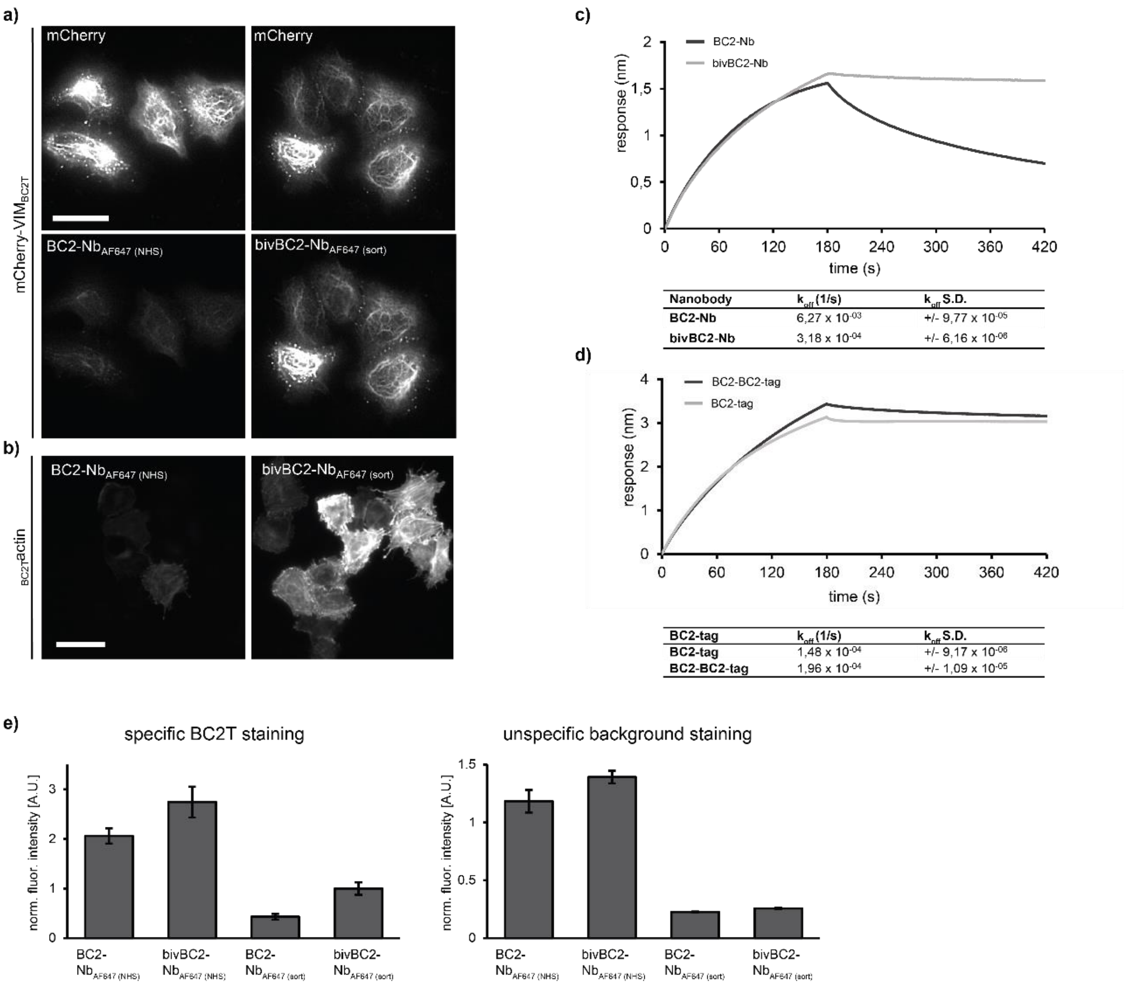

Characterization of binding properties and labeling intensities of monovalent vs. bivalent BC2-Nb. (**a**) Comparison of labeling intensities of mono- and bivalent BC2 nanobodies either labeled via NHS conjugation to Alexa Fluor 647 (BC2-Nb$_{AF647\ (NHS)}$) or sortase-mediated coupling (bivBC2-Nb$_{AF647\ (sort)}$). HeLa cells transiently expressing a C-terminally BC2-tagged mCherry-vimentin construct (mCherry-VIM$_{BC2T}$, upper row) were fixed and stained with either BC2-Nb$_{AF647\ (NHS)}$ (lower row, left panel) or bivBC2-Nb$_{AF647\ (sort)}$ (lower row, right panel). Scale bar, 50 µm. (**b**) Comparison of labeling intensities of BC2-Nb$_{AF647\ (NHS)}$ and bivBC2-Nb$_{AF647\ (sort)}$ on BC2-tagged actin ($_{BC2T}$actin). HeLa cells transiently expressing $_{BC2T}$actin were fixed and stained with either BC2-Nb$_{AF647\ (NHS)}$ (left panel) or bivBC2-Nb$_{AF647\ (sort)}$ (right panel). Scale bar, 50 µm. (**c**) Determination of nanobody binding kinetics by bio-layer interferometry. Exemplary sensograms of BC2-Nb (240 nM) and bivBC2-Nb (120 nM) are shown. The table summarizes

the dissociation rate (k_{off}) and standard deviation (S.D.) of BC2-Nb and bivBC2-Nb derived from the analysis of three concentrations (120 nM, 240 nM, 480 nM). (**d**) Determination of bivBC2-Nb$_{AF647\ (sort)}$ binding kinetics on mono or tandem BC2-tag (BC2-BC2-tag). Exemplary sensograms of bivBC2-Nb$_{AF647\ (sort)}$ (120 nM) are shown. The table summarizes the dissociation rate (k_{off}) and standard deviation (S.D.) of bivBC2-Nb$_{AF647\ (sort)}$ derived from the analysis of three concentrations (120 nM, 240nM, 480nM). (**e**) Assessment of staining quality. Labeling of the different nanobody formats was quantified by measuring the signal intensity of mCherry-VIM$_{BC2T}$ expressing cells (left) and non-transfected cells (background, right), (BC2-Nb$_{AF647\ (NHS)}$: n=115; bivBC2-Nb$_{AF647\ (NHS)}$: n=134; BC2-Nb$_{AF647\ (sort)}$: n=150; bivBC2-Nb$_{AF647\ (NHS)}$: n=195). Calculated ratio is shown in **Fig 1c**.

Supplementary Figure 2

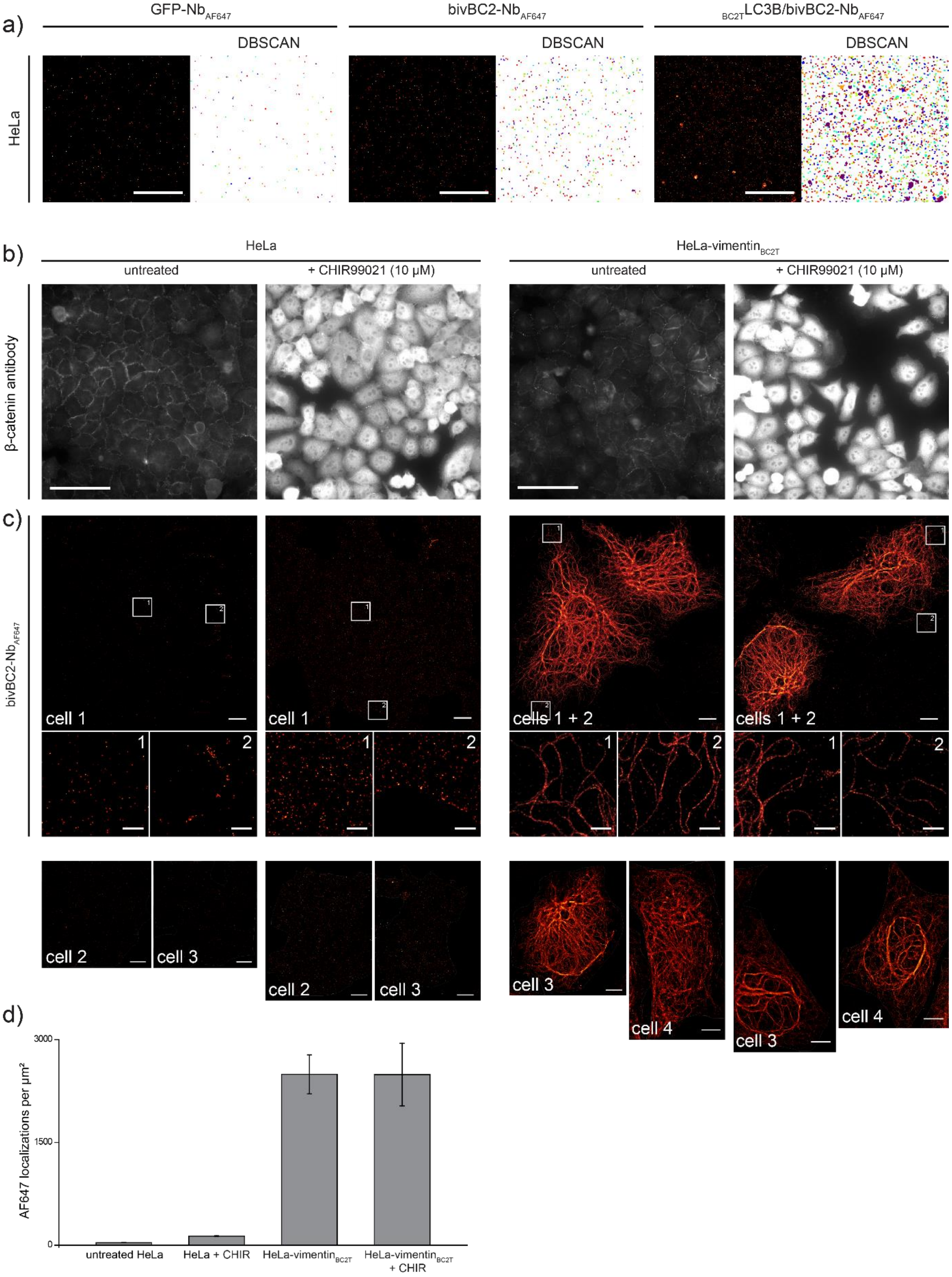

Assessment of bivBC2-Nb$_{AF647}$ staining of endogenous β-catenin (**a**) dSTORM images and corresponding DBSCAN plots, showing only clustered localizations of untransfected

chemically fixed HeLa cells stained with GFP-Nb_{AF647} and bivBC2-Nb_{AF647} , as well chemically fixed HeLa cells transiently expressing $_{BC2T}$LC3B stained with bivBC2-Nb_{AF647} . Scale bars 5 µm. N = 3 cells for each condition. Bar chart summarizing all three conditions is shown in **Fig. 1c**. (**b**) Untransfected HeLa cells (left panel) or HeLa cells expressing C-terminally BC2-tagged vimentin ($vimentin_{BC2T}$) were left untreated or incubated with CHIR99021. Cells were chemically fixed and stained with a conventional anti-β-catenin antibody. Scale bar, 100 µm. (**c**) dSTORM images of cells as described in (**b**) stained with bivBC2-Nb_{AF647}. Scale bars in images 5 µm and 1 µm in insets. Additional dSTORM images used in localization counting analysis (lower panel). (**d**) Localization counts per $µm^2$ plotted as bar charts for all four conditions. Errors are given as standard deviation (S.D.). Image reconstruction details are given in the **Methods** section.

Supplementary Figure 3

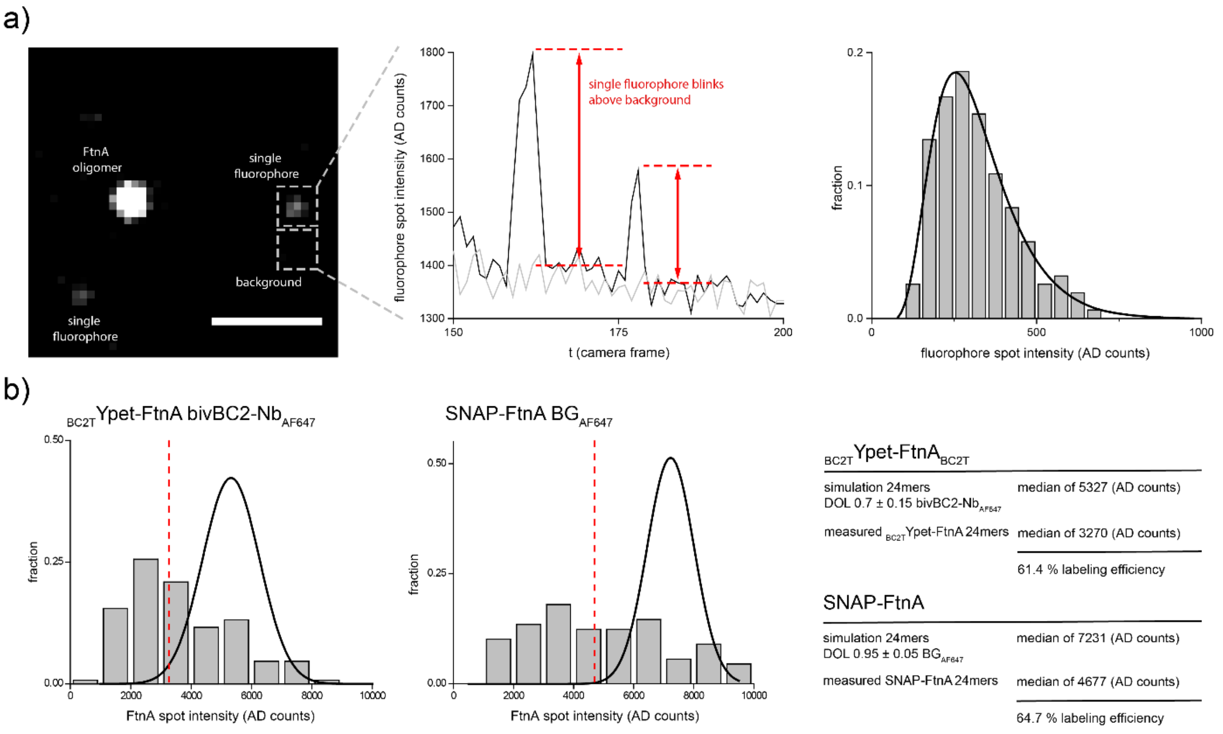

Quantification of completeness of labeling of FtnA oligomers tagged with the BC2- or SNAP-tag. (**a**) Wide-field fluorescence image of a $_{BC2T}$YPET-FtnA oligomer stained with the bivBC2-Nb$_{AF647}$ and single AF647 molecules bound to the slide surface (dashed line). Scale bar 1 µm. Single AF647 molecule intensity trace plotted in integrated intensity AD counts (y axis) over time in frame number (x axis). Blinking events are visible as clear jumps in fluorescence over the background (marker with red arrows and dashed lines). Right panel shows distribution of AF647 single molecule intensities plotted as a relative fraction histogram of integrated intensities with a bin size of 100 AD counts, fitted with a log-normal distribution function. (**b**) Distribution of bivBC2-Nb$_{AF647}$ and AF647-BG stained $_{BC2T}$Ypet-FtnA and SNAP-FtnA spot intensities (in the red channel) plotted as relative fraction histograms of integrated intensity AD counts with a bin size of 1000 AD counts is shown. Red lines represent the median value of both populations and the black curves represent the intensity distribution of simulated theoretical staining maximum. Table summarizes median values of simulations and measured distributions of FtnA oligomers which yields the completeness of labeling for both labeling systems. Corresponding summarizing bar chart is shown in **Fig. 1c**.

Supplementary Figure 4

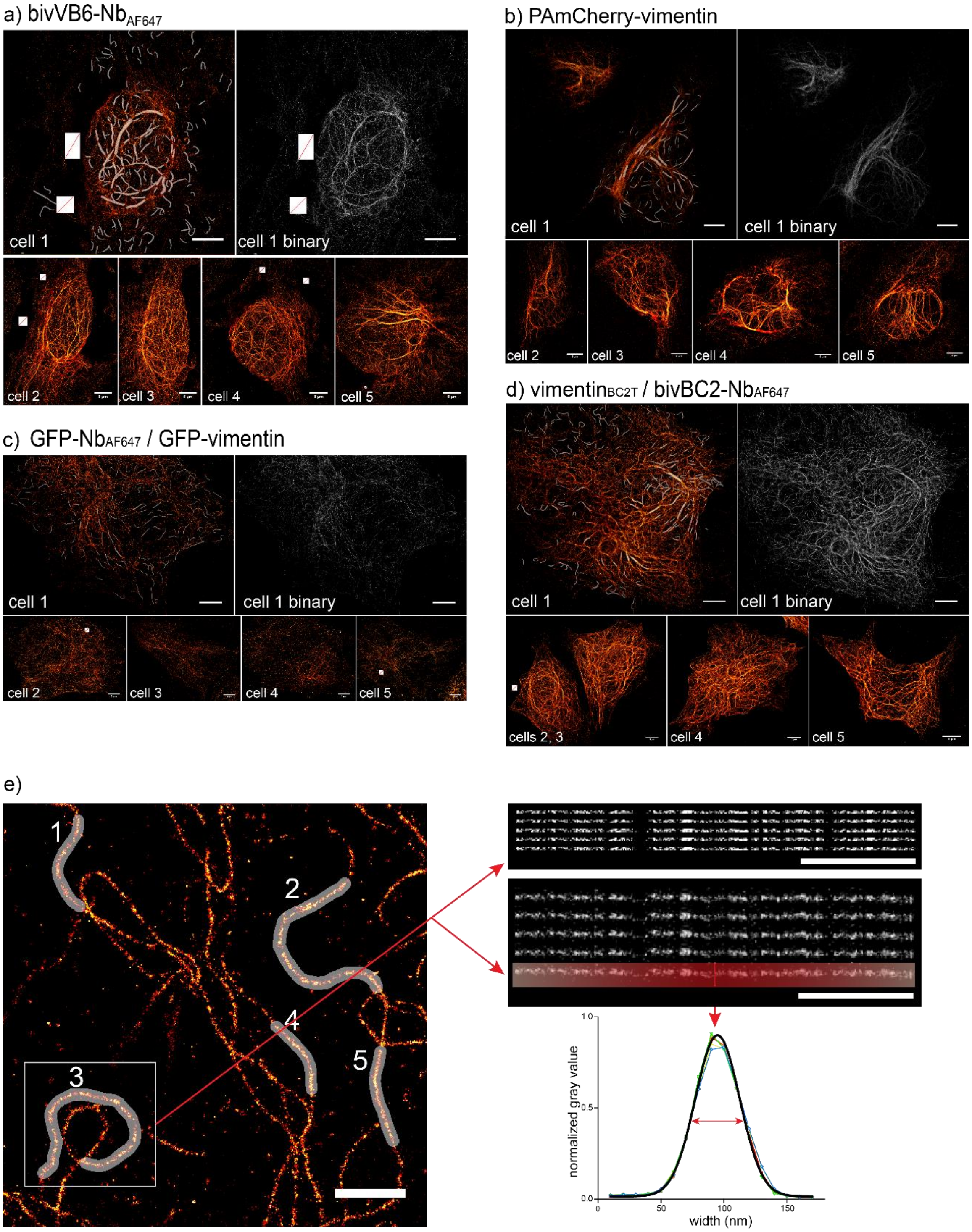

Analysis of PALM/dSTORM images of vimentin filaments in chemically fixed HeLa cells. (**a**) dSTORM images of five cells with native vimentin labeled with bivVB6-Nb$_{AF647}$. (**b**) dSTORM images of five cells expressing GFP-vimentin labeled with GFP-Nb$_{AF647}$. (**c**) dSTORM images of five cells expressing vimentin$_{BC2T}$ labeled with bivBC2-Nb$_{AF647}$. (**d**) PALM images of five cells expressing PAmCherry-vimentin. Scale bars, 5 µm.

Cells were analyzed with a custom-written ImageJ script. Each image was divided into 10 µm x 10 µm sections and 15 filaments per section were analyzed. First images include overlays for all analyzed filaments. All images are calculated corresponding to their individual experimental spatial resolution using the NeNA value. A binary version of each image was used to calculate filament coverages. The workflow is summarized in (**e**); first, lines were drawn along filaments. To minimize the selection and pixilation error, selections were shifted by 0.5 pixels (5 nm) in all directions to obtain five measures in total for each filament by straightening all selections. The middle 3 pixels filaments were taken from the binary image to calculate lengthwise coverage. The coverage of each filament was obtained from averaging the five measurements. To determine the filament width, lengthwise intensity profiles of the five filament selections were fitted with Gaussian curves. The average full width at half maximum (FWHM) of the five selections yields the average filament width. Width and lengthwise fluorophore coverage were analyzed for a total of 676 (bivVB6-Nb_{AF647}), 295 (PAmCherry), 724 (GFP-Nb_{AF647}) and 620 (bivBC2-Nb_{AF647}) filaments as shown in **Fig. 2**. Scale bars, 1 µm. Image reconstruction details are given in the **Methods** section

Supplementary Figure 5

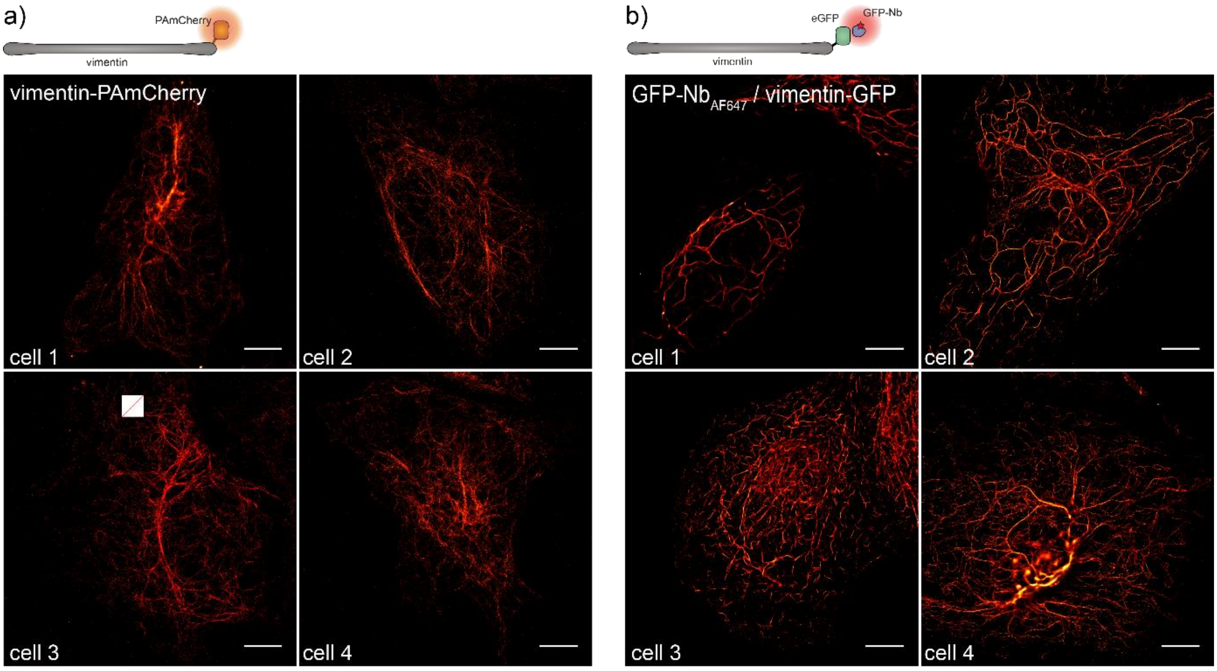

Representative PALM images of chemically fixed HeLa cells transiently expressing (**a**) vimentin C-terminally tagged with photoactivatable mCherry (vimentin-PAmCherry) and (**b**) dSTORM images of chemically fixed HeLa cells transiently expressing vimentin C-terminally with eGFP followed by staining with GFP-Nb$_{AF647}$ are shown. While vimentin-PAmCherry expressing cells are small and show thick vimentin bundles and few thin filaments (cell 1 - 4), vimentin-GFP expressing cells display different phenotypes, from only a few thick filaments (cell 1), networks of uniform medium-thick filaments (cell 2), fragmented filaments (cell 3) and very few cells that appear physiological (cell 4). Scale bars, 5 µm. Image reconstruction details are given in the **Methods** section.

Supplementary Figure 6

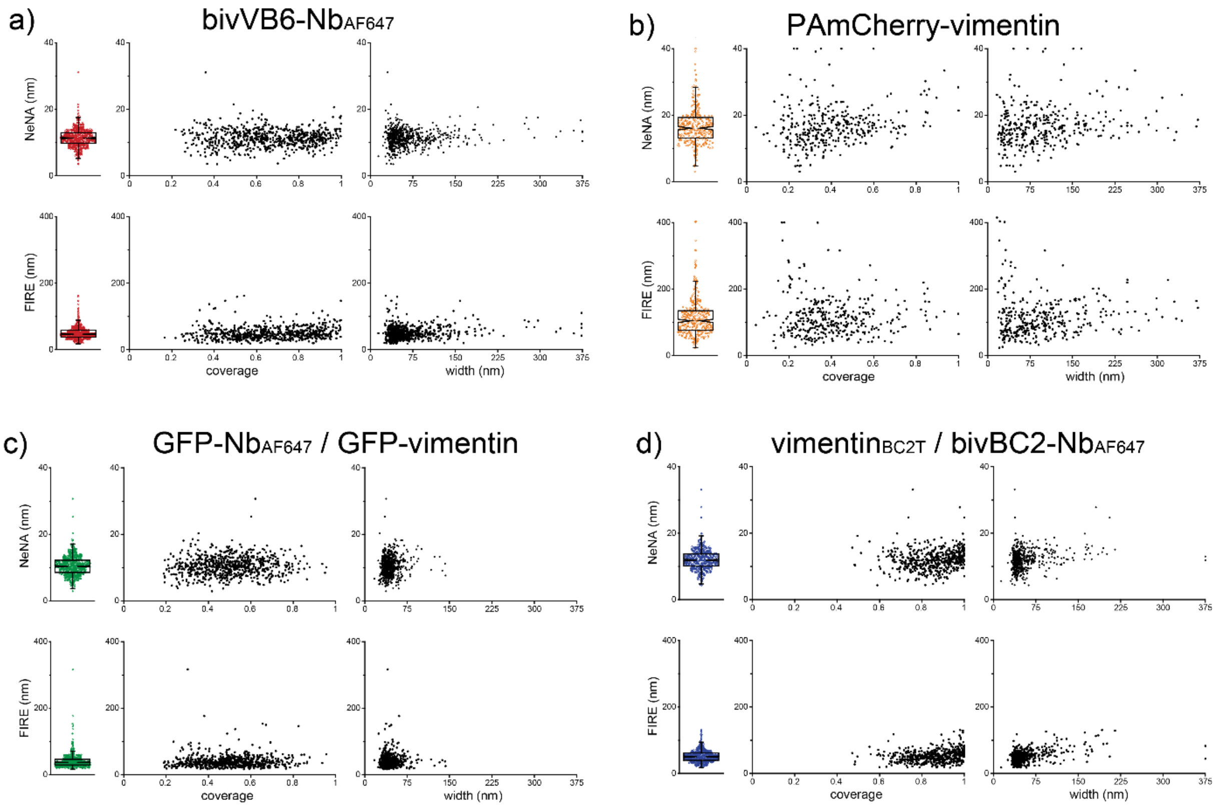

Plots of NeNA and FIRE image resolution analysis for PALM/dSTORM images. Graphs are shown for (**a**) native vimentin labeled with bivVB6-Nb$_{AF647,}$ (**b**) GFP-vimentin labeled with GFP-Nb$_{AF647}$ (**c**) vimentin$_{BC2T}$ labeled with bivBC2-Nb$_{AF647}$ (**d**) PAmCherry-vimentin. Scatter + box plots (the box marks the 3 quartiles and the whiskers mark 95% of all the data) of all calculated NeNA (upper) and FIRE (lower) values as individual filament measurements. Individual NeNA and FIRE values were also plotted against filament coverage (middle, in fraction) and filament width (left, in nm) for each condition. The total number of filament ROIs was 636 (NeNA bivVB6$_{AF647}$), 644 (FIRE bivVB6$_{AF647}$), 347 (NeNA PAmCherry), 353 (FIRE pPAmCherry), 714 (NeNA bivVB6$_{AF647}$), 682 (FIRE bivVB6$_{AF647}$), 514 (NeNA bivBC2-Nb$_{AF647}$) and 519 (FIRE bivBC2-Nb$_{AF647}$). Numbers differ slightly from the total n of chosen ROIs as for some ROIs no NeNA or FIRE value could be calculated.

Supplementary Figure 7

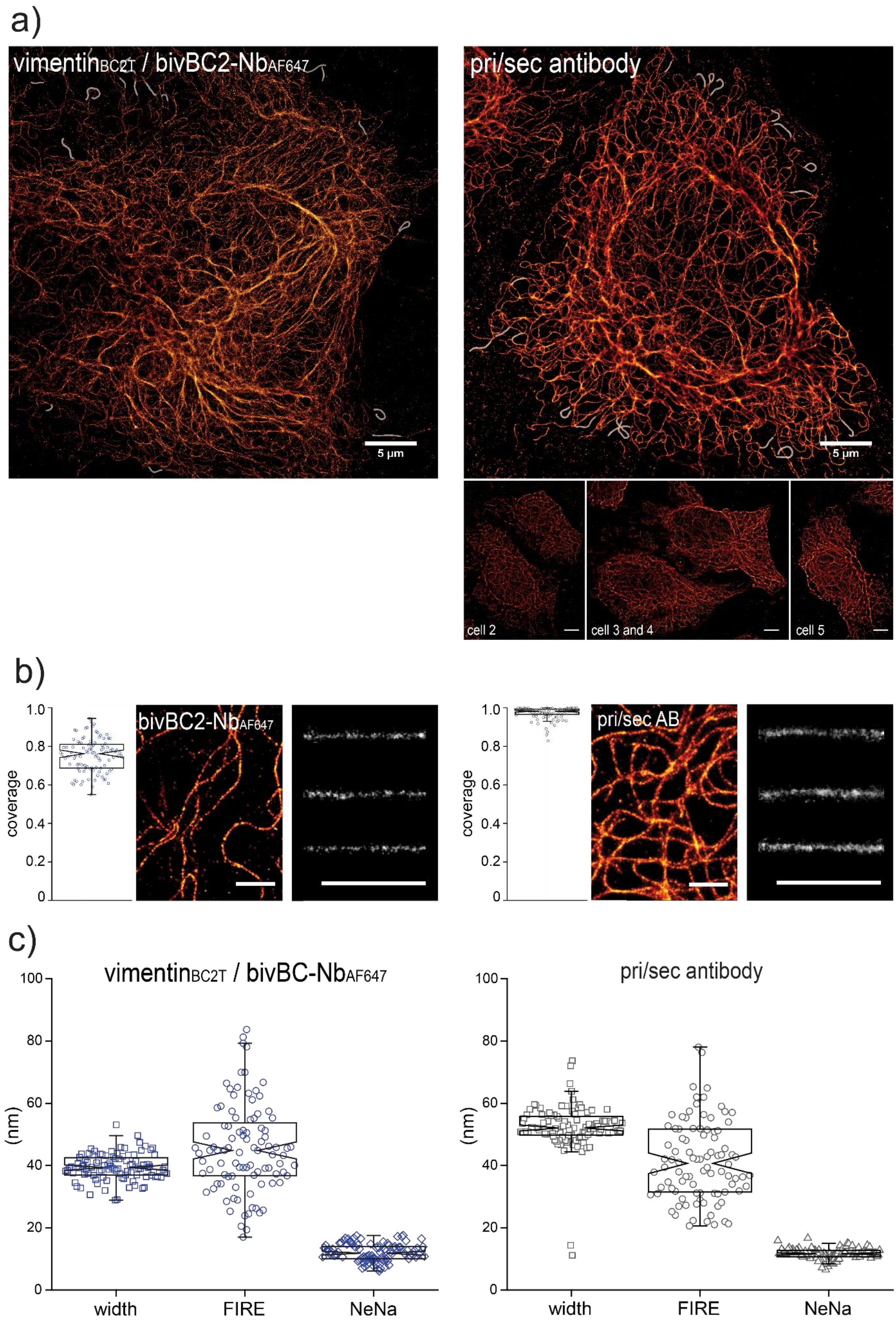

bivBC2-Nb labeling of BC2-tagged vimentin compared to conventional antibody labeling of native vimentin. (**a**) Representative dSTORM images of chemically fixed HeLa cells expressing $vimentin_{BC2T}$ stained with bivBC2-Nb_{AF647} or chemically fixed HeLa cells where native vimentin was stained with a conventional primary antibody followed by staining with a secondary antibody coupled to AF647. Scale bars, 5 µm. 100 peripheral (single) filaments were analyzed per labeling strategy. (**b**) Coverage analyses of affinity tags. Scatter + box plots (the box marks the 3 quartiles and the whiskers mark 95% of all the data.) of thin filament coverages for bivBC2-Nb_{AF647} and pri/sec antibody staining plotted as individual filament statistics of fluorophore covered fractions and representative peripheral filaments, before analysis and straightened. Scale bars, 1 µm. 100 filaments were analyzed per method. (**c**) Scatter + box plots (descriptive statistics same as (**b**) of thin filament widths, FIRE values and NeNA values for all conditions. Image reconstruction details are given in the **Methods** section.

Supplementary Figure 8

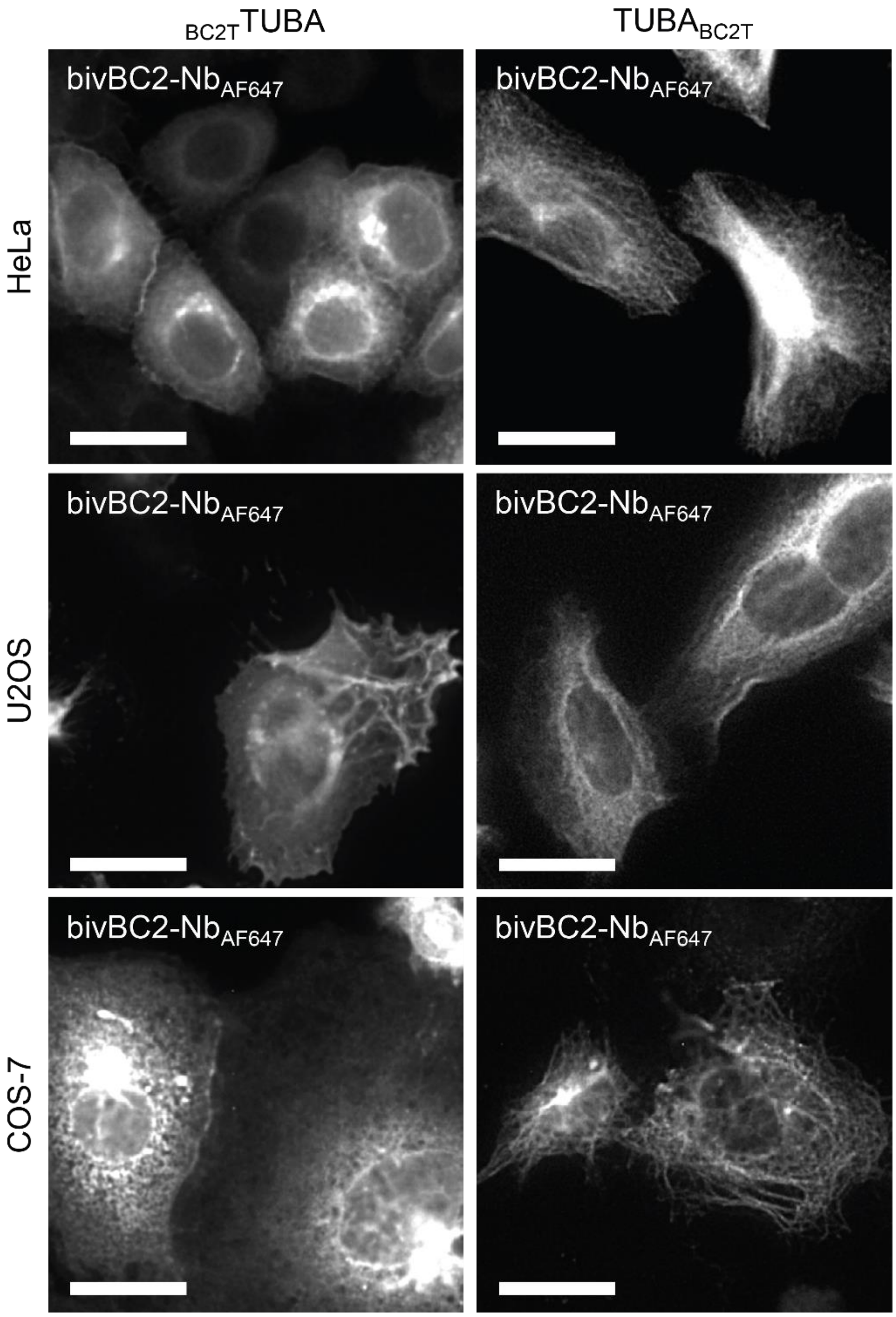

Visualization of N- or C-terminally BC2-tagged tubulin-alpha-1B chain (*TUBA*) in human cells. Immunofluorescence images of chemically fixed HeLa, U2OS and COS-7 cells transiently expressing N- or C-terminally BC2-tagged tubulin ($_{BC2T}$TUBA, TUBA$_{BC2T}$). Cells were stained with the bivBC2-Nb$_{AF647}$. Scale bars, 25 µM.

Supplementary Figure 9

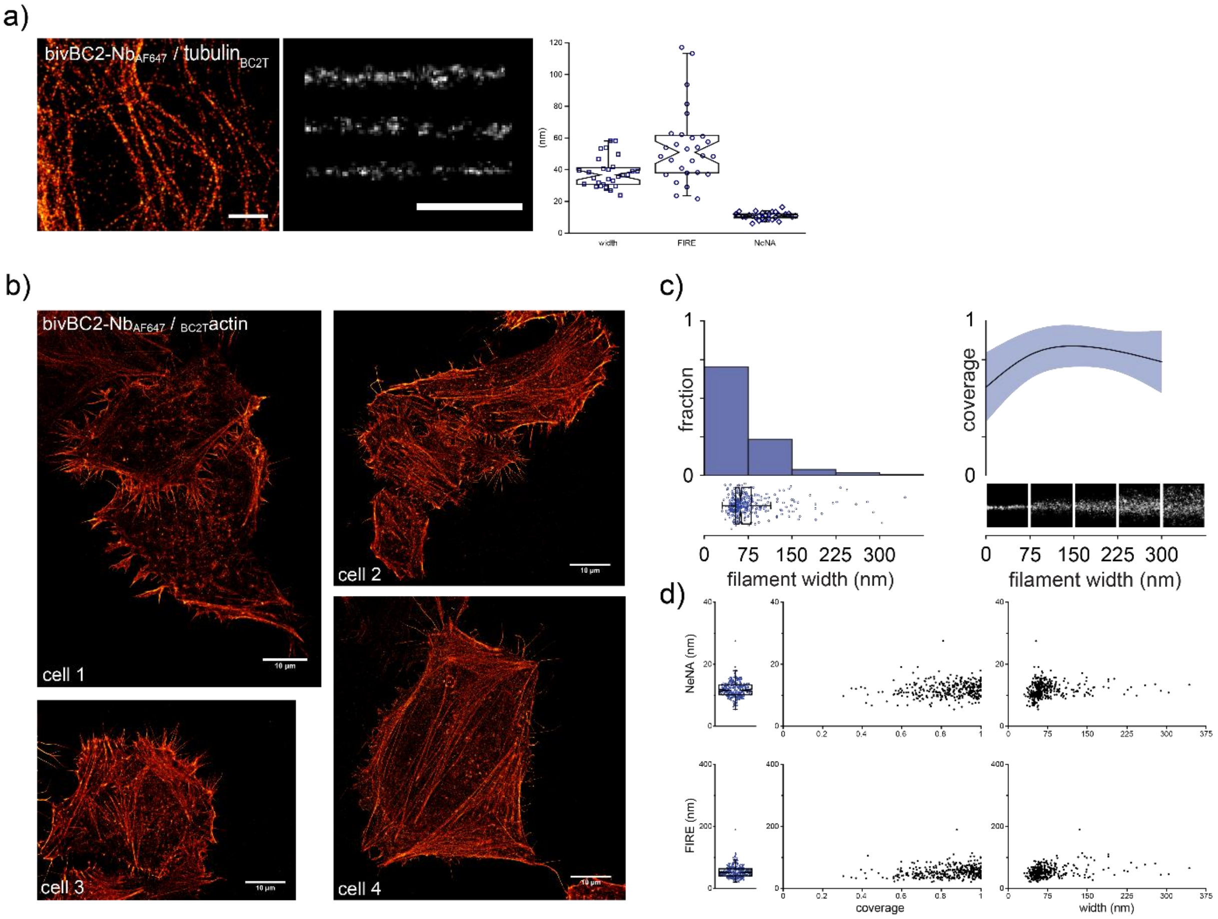

Image resolution measures for tubulin and actin visualized by tubulin$_{BC2T}$ or $_{BC2T}$actin respectively. (**a**) Tubulin fibers of an exemplary HeLa cell expressing tubulin$_{BC2T}$ are straightened like for vimentin in **Supplementary Fig. 4** and **6** and analyzed for filament width yielding a FWHM of 38.2 ± 9.2 nm. (n = 29 fibers). (**b**) dSTORM images of five chemically fixed cells expressing $_{BC2T}$actin labeled with bivBC2-Nb$_{AF647}$. Scale bars, 10 µm. (**c**) Actin filament widths as histograms (left) with a bin size of 75 nm (x axis) plotted against relative fraction (y axis). Full data is represented underneath the histograms as box + scatter plots with the same x axis. The box marks the 3 quartiles and the whiskers mark 95% of all the data. The average lengthwise fluorophore coverage was calculated for each bin and plotted (right) as mean filament width (black line) and standard deviation (colored area) against relative fraction covered by fluorophores (y axis). A total of 351 filaments were analyzed for width and lengthwise fluorophore coverage. (**d**) Scatter + box plots of all calculated NeNA (upper) and FIRE (lower) as individual filament measurements. Individual NeNA and FIRE values were

also plotted against filament coverage (middle, in fraction) and filament width (left, in nm). (Sample size same as in (**c**)). Image reconstruction details are given in the **Methods** section.

Supplementary Figure 10

a)

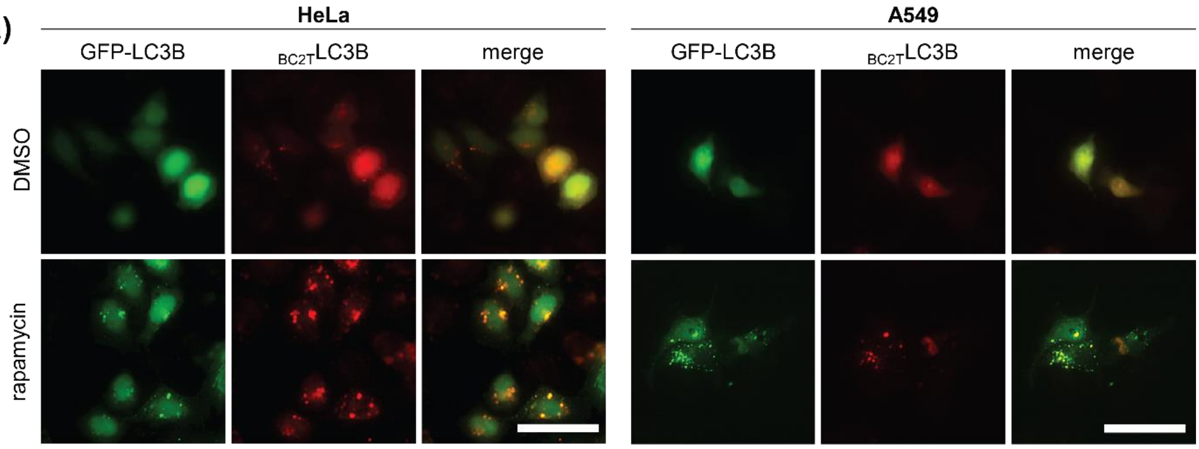

b)

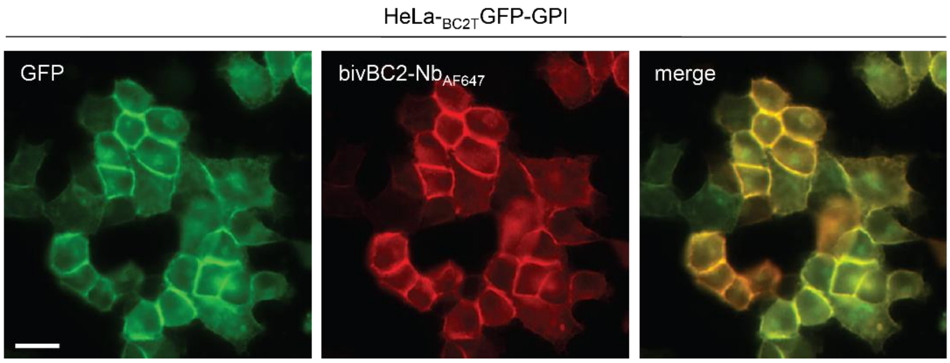

Detection of non-structural proteins with the BC2-tag/bivBC2-Nb system. (**a**) HeLa cells or A549 cells transiently coexpressing GFP-LC3B and $_{BC2T}$LC3B were incubated with 0.5 µM rapamycin to induce autophagy or control treated with 0.1 % DMSO. Shown are images of chemically fixed cells displaying the co-localizing GFP- and bivBC2-Nb$_{AF647}$ signal after 20 h incubation with rapamycin. (**b**) HeLa cells expressing $_{BC2T}$GFP-GPI were chemically fixed and stained with the bivBC2-Nb$_{AF647}$. Shown are representative images displaying co-localizing GFP- and bivBC2-Nb$_{AF647}$ signals at the plasma membrane. Scale bars, 25 µm.

Supplementary Figure 11

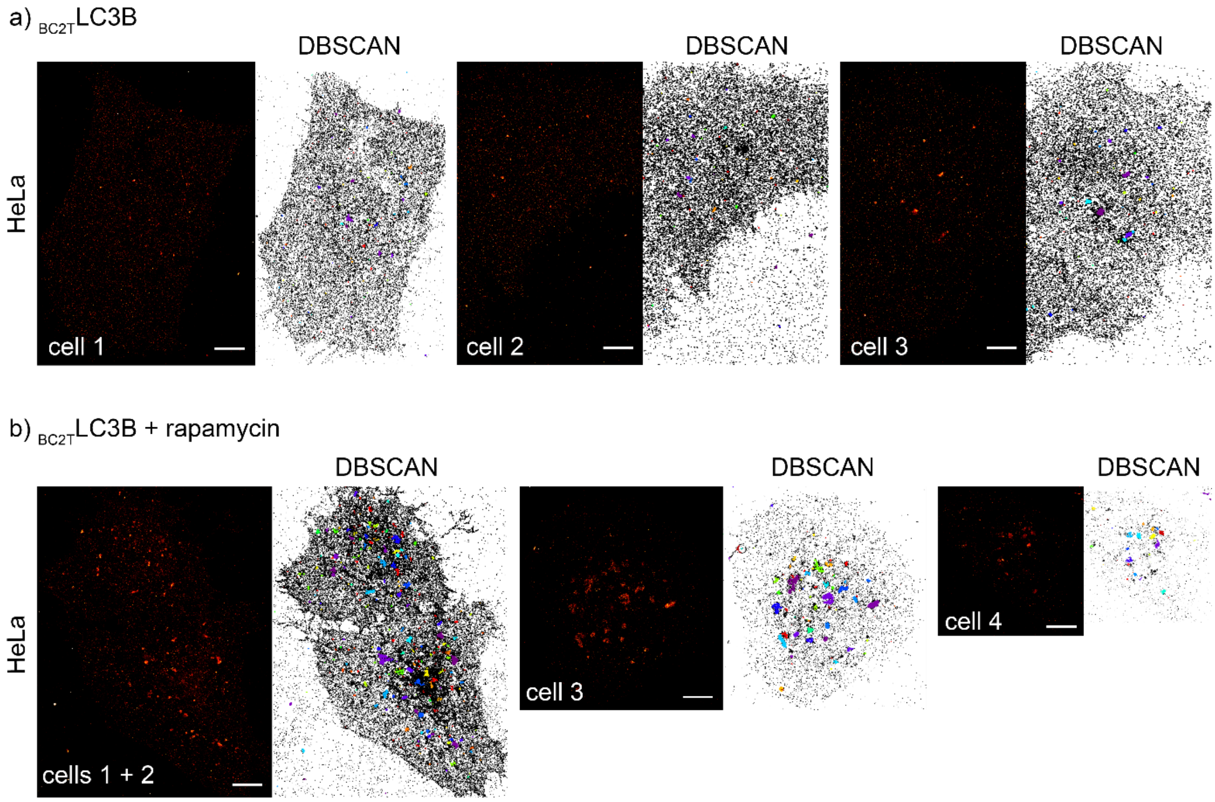

dSTORM images (left) and corresponding DBSCAN plots (right) of all the chemically fixed HeLa cells transiently expressing $_{BC2T}$LC3B that were used for analysis shown in **Fig. 3d**. (**a**) non-treated cells, n = 120 clusters for cell 1, 76 clusters for cell 2 and 146 clusters for cell 3. (**b**) cells treated with rapamycin, n = 203 clusters for cell 1, 74 for cell 2, 85 for cell 2 and 43 for cell 4. Image reconstruction details are given in the **Methods** section. Summarizing bar charts are shown in **Fig. 3d**.

Supplementary Figure 12

a)

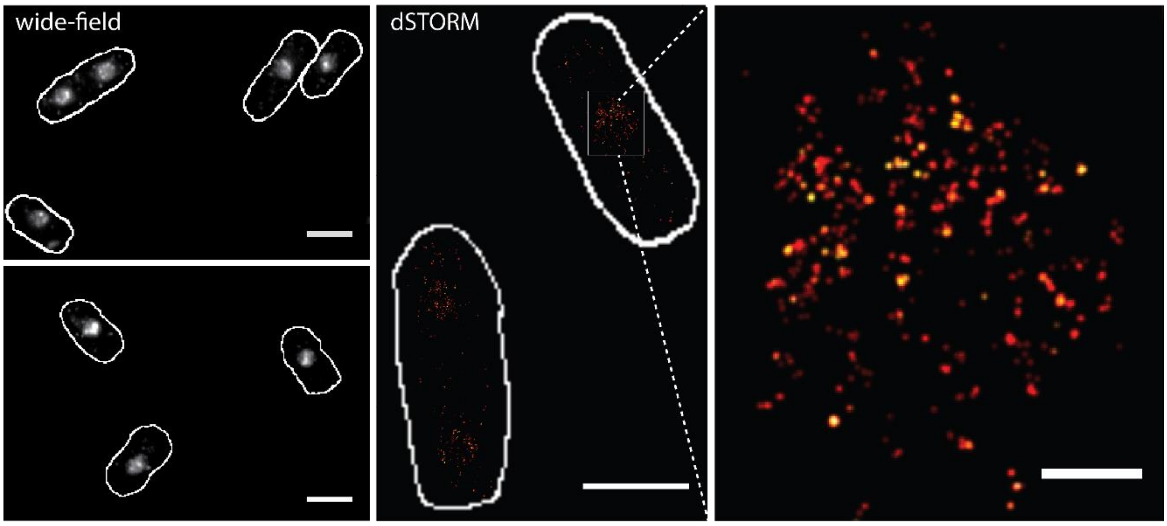

b)

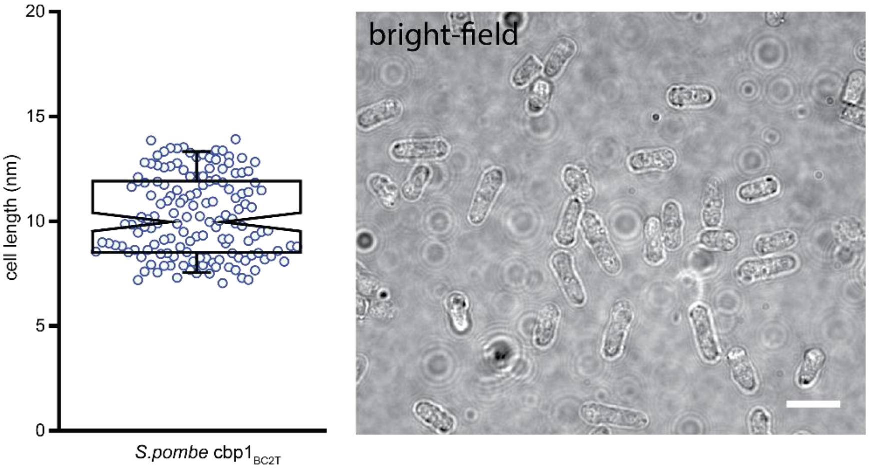

Labeling of the endogenously tagged DNA-binding protein cbp1 in *S. pombe.* (**a**) Shown are exemplary wide-field images of C-terminally tagged cbp1 ($cbp1_{BC2T}$) in chemically fixed *S. pombe* cells, scale bar, 10 µm (left panel). The nuclear $cbp1_{BC2T}$ was then imaged by dSTORM to resolve the distribution of individual proteins within the nucleus, scale bar, 5 µm (inset, scale bar, 0.5 µm). (**b**) The *S. pombe* strain encoding $cbp1_{BC2T}$ at the endogenous locus does not show any growth defects when analyzing the cell length distribution, n = 100 cells, nor abnormalities in the phenotype. Scale bar, 10 µm. Image reconstruction details are given in the **Methods** section.

Supplementary Figure 13

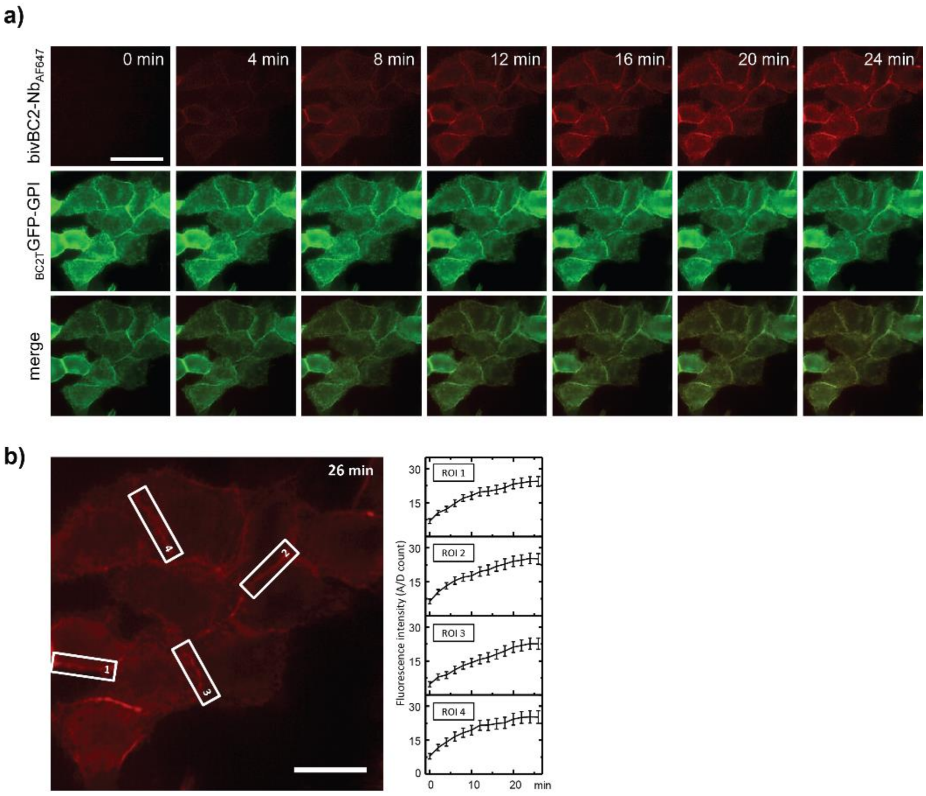

Imaging of BC2-tagged membrane protein in living cells. (**a**) HeLa cells expressing $_{BC2T}$GFP-GPI were subjected to live cell imaging 72 h after transfection. Shown are images displaying the nanobody signal (upper row) and GFP-signal (mid-row) derived from time lapse acquisition starting with the addition of bivBC2-Nb$_{AF647}$. Scale bar, 50 µm. (**b**) The fluorescence staining process was assessed by quantifying the fluorescence intensity increase over time for four different regions of interest (ROI 1 - 4, left) of cellular contact zones. Plotted (right) are the mean intensity values of each ROI and s.e.m. error bars. Scale bar 25 µm.

Supplementary Figure 14

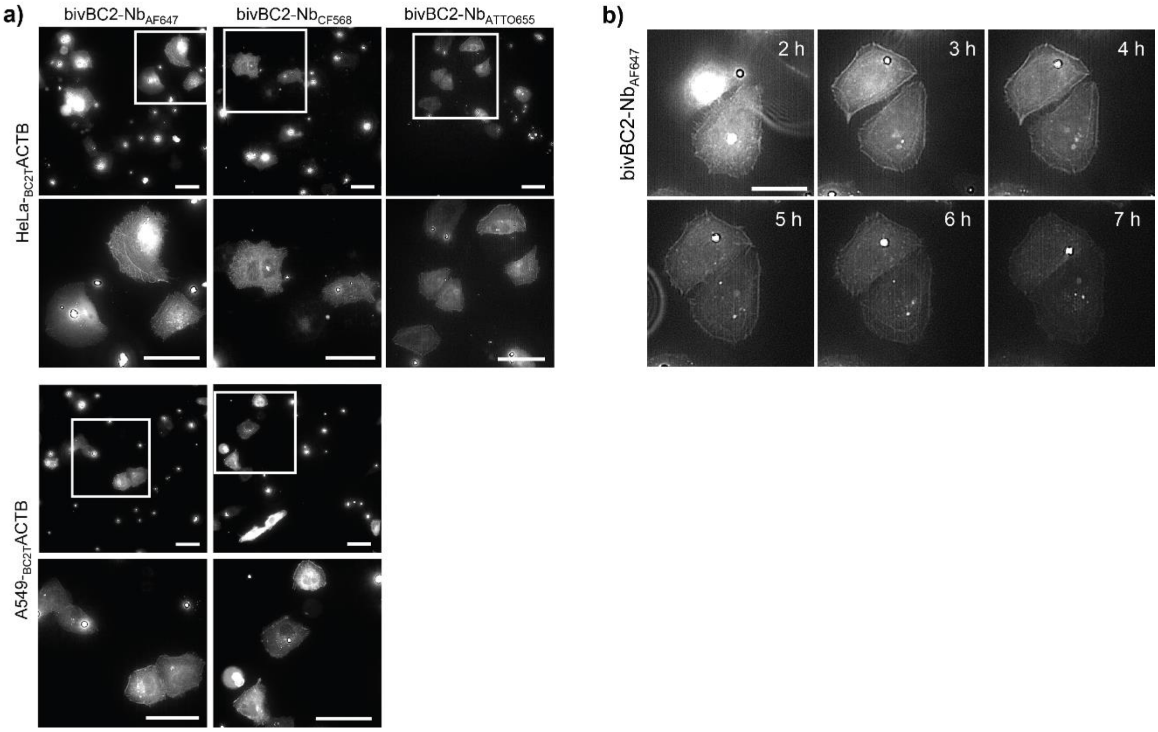

(**a**) Labeling of endogenous BC2-tagged actin upon transduction of bivBC2-Nb conjugated to different fluorescent dyes. Shown are representative images of living HeLa-$_{BC2T}$ACTB cells labeled either with bivBC2-Nb$_{AF647}$, bivBC2-Nb$_{CF568}$ or bivBC2-Nb$_{ATTO655}$ (upper panel) and A549-$_{BC2T}$ACTB cells labeled with either bivBC2-Nb$_{AF647}$ or bivBC2-Nb$_{CF568}$ (lower panel). Selected areas of transduced cells (indicated by white squares) are depicted as enlarged images. Scale bars, 50 µM. (**b**) Time lapse imaging of HeLa-$_{BCT2}$ACTB upon transduction of the bivBC2-Nb$_{AF647}$. Shown are representative images of two nanobody-transduced cells. Scale bar, 25 µm.

Supplementary Figure 15

a) chemically fixed HeLa-$_{BC2T}$ACTB/bivBC2-Nb$_{AF647}$

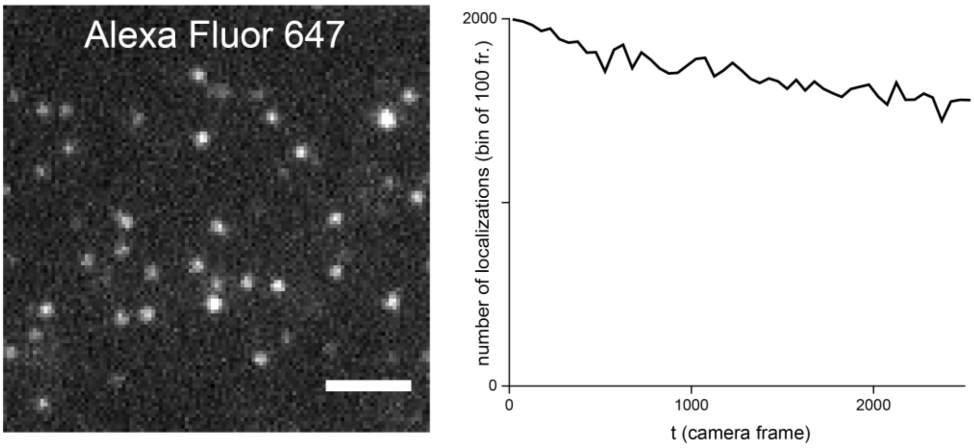

b) living HeLa-$_{BC2T}$ACTB/bivBC2-Nb$_{ATTO655}$

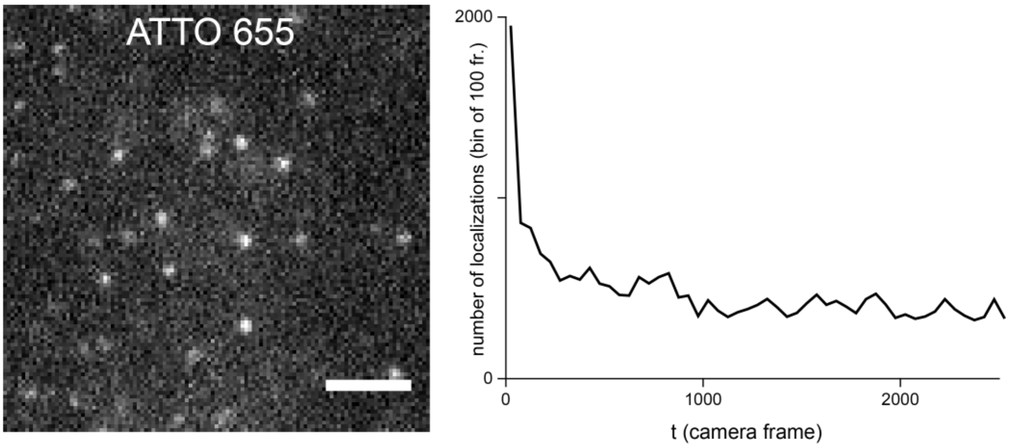

Comparison of ATTO655 and AF647 photophysics under dSTORM imaging conditions in living and chemically fixed cells. (**a**) Representative single imaging frame, extracted from a dSTORM movie of chemically fixed HeLa-$_{BC2T}$ACTB stained with the bivBC2-Nb$_{AF647}$, under standard dSTORM conditions (**Methods**). Line graph representing the absolute frequency count of localizations (y axis) at a bin size of 100 over the first 2500 frames of a 15 x 15 µm ROI of the movie (x axis). (**b**) Representative single imaging frame, extracted from a dSTORM movie of live HeLa-$_{BC2T}$ACTB stained with the bivBC2-Nb$_{ATTO655}$. Line graph representing the absolute frequency count of localizations (y axis) at a bin size of 100 over the first 2500 frames of a 15 x 15 µm ROI of the movie (x axis). Imaging sequences taken from raw data acquisitions of both conditions can be found in **Supplementary Movies 5 and 6.**

References

1. Nieuwenhuizen, R.P. et al. Measuring image resolution in optical nanoscopy. *Nature methods* **10**, 557-562 (2013).
2. Braun, M.B. et al. Peptides in headlock–a novel high-affinity and versatile peptide-binding nanobody for proteomics and microscopy. *Scientific reports* **6** (2016).
