# Supplementary material for: A peptide tag-specific nanobody enables high-quality labeling for dSTORM imaging
Source: Nat Commun. 2018 Mar 2;9:930. doi: 10.1038/s41467-018-03191-2 (PMC5834503; doi:10.1038/s41467-018-03191-2)
Supplement: Supplementary file 3 — Description of Additional Supplementary Files [file 41467_2018_3191_MOESM3_ESM.pdf]

Description of Additional Supplementary Files

File Name: Supplementary Movie 1
Description: Imaging sequence taken from raw data acquisition of the BC2TGFP-GPI dynamics of the HeLa cell shown in Fig.5a.

File Name: Supplementary Movie 2
Description: Imaging sequence taken from raw data acquisition of the BC2TGFP-GPI dynamics of the inset 1 from the HeLa cell shown in Fig.5a.

File Name: Supplementary Movie 3
Description: Imaging sequence taken from raw data acquisition of the BC2TGFP-GPI dynamics of the inset 2 from the HeLa cell shown in Fig.5a.

File Name: Supplementary Movie 4
Description: Imaging sequence taken from raw data acquisition of the BC2TGFP-GPI dynamics of the inset 3 from the HeLa cell shown in Fig.5a.

File Name: Supplementary Movie 5
Description: Imaging sequence taken from raw data acquisition of the chemically fixed HeLa-BC2TACTB cell shown in Fig.4b visualizing the brightness and photoswitching characteristics of AF647 fluorophores. Further assessment of AF647 photophysics under dSTORM imaging conditions can be found in Supplementary Fig. 15.

File Name: Supplementary Movie 6
Description: Imaging sequence taken from raw data acquisition of the living HeLa-BC2TACTB cell shown in Fig.5b visualizing the brightness and photoswitching characteristics of ATTO655 fluorophores. Further assessment of ATTO655 photophysics under live cell dSTORM imaging conditions can be found in Supplementary Fig. 15.
