# Supplementary figures and images for: A peptide tag-specific nanobody enables high-quality labeling for dSTORM imaging
Source: Nat Commun. 2018 Mar 2;9:930. doi: 10.1038/s41467-018-03191-2 (PMC5834503; doi:10.1038/s41467-018-03191-2)

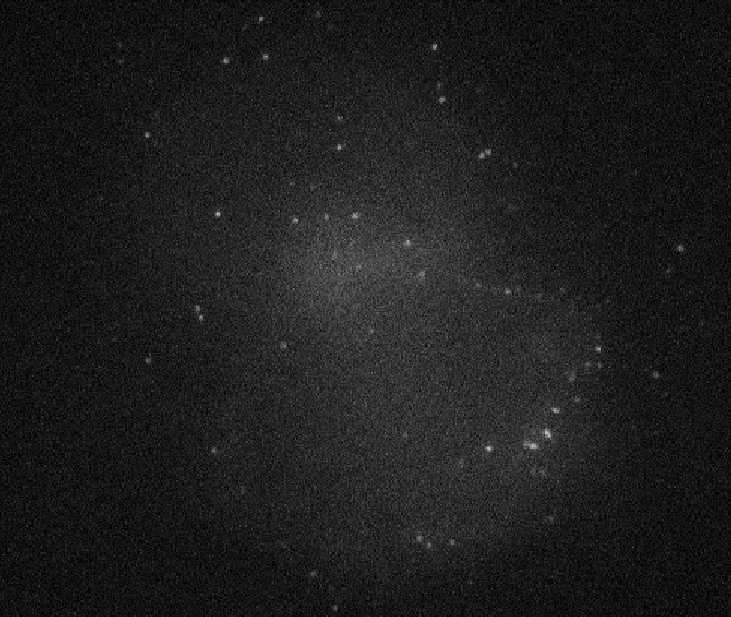

Supplement: Supplementary file 4 — Supplementary Movie 1 [file 41467_2018_3191_MOESM4_ESM.gif]

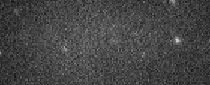

Supplement: Supplementary file 5 — Supplementary Movie 2 [file 41467_2018_3191_MOESM5_ESM.gif]

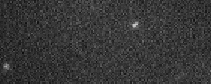

Supplement: Supplementary file 6 — Supplementary Movie 3 [file 41467_2018_3191_MOESM6_ESM.gif]

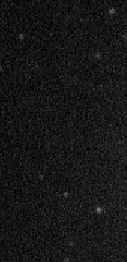

Supplement: Supplementary file 7 — Supplementary Movie 4 [file 41467_2018_3191_MOESM7_ESM.gif]

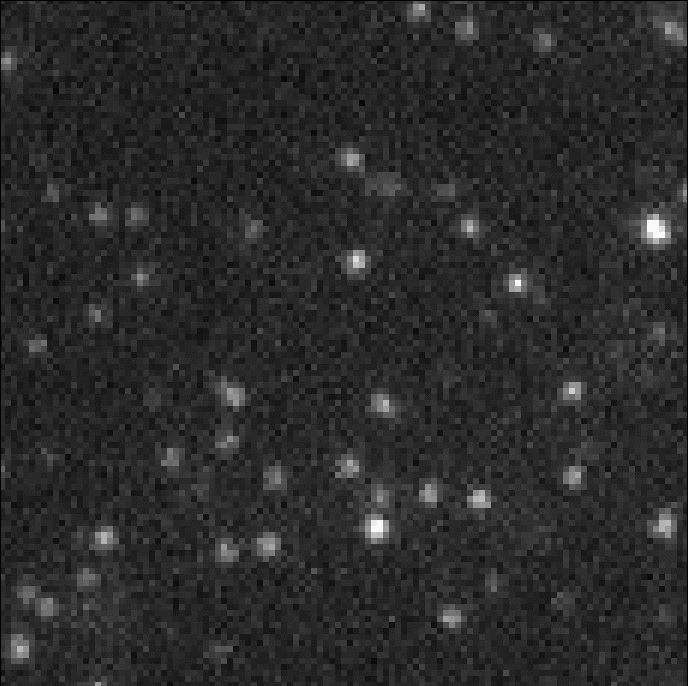

Supplement: Supplementary file 8 — Supplementary Movie 5 [file 41467_2018_3191_MOESM8_ESM.gif]

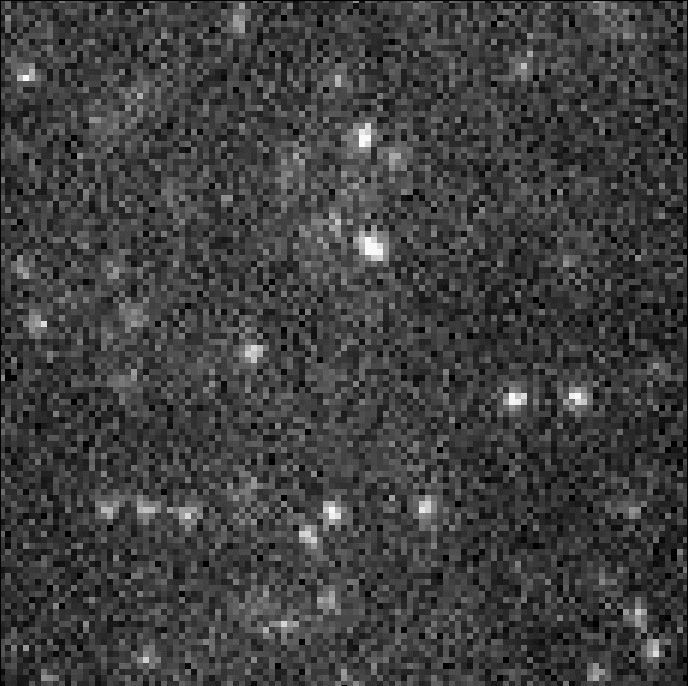

Supplement: Supplementary file 9 — Supplementary Movie 6 [file 41467_2018_3191_MOESM9_ESM.gif]
